# MicroRNAs as early toxicity signatures of doxorubicin in human-induced pluripotent stem cell-derived cardiomyocytes

**DOI:** 10.1007/s00204-016-1668-0

**Published:** 2016-02-03

**Authors:** Umesh Chaudhari, Harshal Nemade, John Antonydas Gaspar, Jürgen Hescheler, Jan G. Hengstler, Agapios Sachinidis

**Affiliations:** 1 Institute of Neurophysiology, Center for Molecular Medicine Cologne (CMMC), University of Cologne, 50931 Cologne, Germany; 2 Leibniz Research Centre for Working Environment and Human Factors at the Technical University of Dortmund (IfADo), 44139 Dortmund, Germany

**Keywords:** Anthracyclines, miRNAs, Cardiotoxicity, Genomic biomarkers

## Abstract

An in depth investigation at the genomic level is needed to identify early human-relevant cardiotoxicity biomarkers that are induced by drugs and environmental toxicants. The main objective of this study was to investigate the role of microRNAs (miRNAs) as cardiotoxicity biomarkers using human-induced pluripotent stem cell (hiPSC)-derived cardiomyocytes (CMs) that were exposed to doxorubicin (DOX) as a “gold standard” cardiotoxicant. hiPSC-CMs were exposed to 156 nM DOX for 2 days or for 6 days of repeated exposure, followed by drug washout and incubation in drug-free culture medium up to day 14 after the onset of exposure. The induced miRNAs were profiled using miRNA microarrays, and the analysis of the data was performed using the miRWalk 2.0 and DAVID bioinformatics tools. DOX induced early deregulation of 14 miRNAs (10 up-regulated and 4 down-regulated) and persistent up-regulation of 5 miRNAs during drug washout. Computational miRNA gene target predictions suggested that several DOX-responsive miRNAs might regulate the mRNA expression of genes involved in cardiac contractile function. The hiPSC-CMs exposed to DOX in a range from 39 to 156 nM did not show a significant release of the cytotoxicity marker lactate dehydrogenase (LDH) compared to controls. Quantitative real-time PCR analyses confirmed the early deregulation of miR-187-3p, miR-182-5p, miR-486-3p, miR-486-5p, miR-34a-3p, miR-4423-3p, miR-34c-3p, miR-34c-5p and miR-1303, and also the prolonged up-regulation of miR-182-5p, miR-4423-3p and miR-34c-5p. Thus, we identified and validated miRNAs showing differential DOX-responsive expression before the occurrence of cytotoxicity markers such as LDH, and these miRNAs also demonstrated the significant involvement in heart failure in patients and animal models. These results suggest that the DOX-induced deregulated miRNAs in human CMs may be used as early sensitive cardiotoxicity biomarkers for screening potential drugs and environmental cardiotoxicants with a similar mechanism of action.

**Electronic supplementary material:**

The online version of this article (doi:10.1007/s00204-016-1668-0) contains supplementary material, which is available to authorized users.

## Introduction

Irreversible cardiac injury is a most serious side effect associated with many known anti-cancer drugs and other therapeutic drugs, putting patients’ lives at risk. Furthermore, over the last two decades, the pharmaceutical industry has been struggling with the costly withdrawals of new drug candidates in late-stage clinical trials. Thus, one of the major tasks of pharmaceutical companies is to predict the cardiotoxic side effects of new drug candidates early in non-clinical phases of drug development. Moreover, it has been recognized since long time that environmental toxicants and pollutants may induce cardiotoxicity in animals and humans (Simkhovich et al. 2009). Several preclinical models have been developed to understand doxorubicin (DOX)-induced early and chronic cardiotoxicity (AbdelAleem et al. 1997; Banco et al. 2011; Desai et al. 2013; Hrelia et al. 2002; Jaenke 1974; Tokarska-Schlattner et al. 2005). Importantly, all of these established models are non-human in origin. Due to an inter-species variation in physiology, assays using these established models cannot reliably predict the cardiotoxic effects of new drugs in humans. However, current studies have demonstrated that in vitro use of human embryonic stem cell (hESC)- or human-induced pluripotent stem cell (hiPSC)-derived cardiomyocytes can be beneficial for preclinical safety assessment.

Currently applied diagnostic techniques [ecocardiographic left ventricular ejection fraction (LVEF), radionuclide angiography and endomyocardial biopsy] for assessing drug-induced cardiac injury have many shortcomings, including sensitivity, invasiveness and high cost. In preclinical and clinical phase studies, troponins (cTnT and cTnI) have been used as highly sensitive plasma biomarkers for detecting DOX-induced cardiac damage (Herman et al. 1998, 1999; Lipshultz et al. 2012; O’Brien 2006; O’Brien et al. 2006; Shahzadi et al. 2014). However, troponins and many other cardiac biomarkers show increased plasma levels only after heart tissue damage and also have a limited half-life (Tonomura et al. 2009; Walker 2006). Considering the disadvantages of current biomarkers, there is an urgent need to identify stable, highly sensitive and noninvasive genomic biomarkers to predict early events of drug-induced cardiac damage.

Among anthracyclines, DOX is a highly effective anti-cancer drug prescribed for the treatment of a variety of cancer types, including solid tumours and haematologic malignancies in both adults and children. Despite its beneficial therapeutic effects, the long-term clinical use of DOX is limited, due to its cumulative dose-dependent cardiotoxicity (Singal and Iliskovic 1998). A recent meta-analysis demonstrates that anthracycline treatment increased the risk of cardiac death by 4.94-fold compared to non-anthracycline treatments (Smith et al. 2010). The acute adverse effects of DOX occur within or after 2–3 days of administration and are manifested by cardiac arrhythmias and acute heart failure. The chronic side effects of DOX are largely dose-dependent. A patient may develop dilated cardiomyopathy shortly after DOX treatment termination, or dilated cardiomyopathy may occur even 10–15 years after the termination of chemotherapy. Acute and chronic DOX administration may lead to cardiac dysfunction, cardiomyopathy and ultimately to heart failure and death (Chatterjee et al. 2010; Wallace 2003; Yeh et al. 2004). DOX is a well-characterized cardiotoxicant of the anthracycline family, but the exact mechanisms of DOX-induced cardiotoxicity are not fully understood. Nevertheless, DOX can be applied as a substance to identify human-relevant genomic biomarkers for potential cardiotoxic drugs and environmental factors that induce cardiotoxicity (Chaudhari et al. 2015).

Small non-coding RNAs that are approximately 22 nucleotides long are known as microRNAs (miRNAs) that regulate gene expression by targeting messenger RNAs (mRNAs) by binding to complementary regions of transcripts to inhibit their translation and/or promote mRNA degradation (Ambros 2004; Bartel 2004). In particular, miRNAs are significantly involved in heart development, physiology and pathogenesis (Latronico and Condorelli 2009; Rao et al. 2009; Small et al. 2010a). These functions make miRNAs an attractive target for identifying sensitive biomarkers of cardiovascular diseases (Bernardo et al. 2012; D’Alessandra et al. 2010; Matsumoto et al. 2013).

Here, for the first time, we identify set of miRNAs as toxicity signatures of DOX in hiPSC-cardiomyocytes (CMs). In the present study, we aimed to identify early and prolonged differential expression of miRNAs in response to DOX exposure in hiPSC-CMs by profiling global miRNA levels. Our miRNA microarray results identified 14 early DOX-responsive miRNAs (10 up-regulated and 4 down-regulated), and five miRNAs that are persistently up-regulated during drug washout. DOX-deregulated miRNAs and their in silico predicted gene targets are associated with intact cardiac function and cardiac diseases. Our results demonstrate that early and/or prolonged deregulated miRNAs are promising broad biomarkers of cardiotoxicity that also provide information regarding the early events of cardiac damage.

## Materials and methods

### Cardiomyocyte cell culture

All experiments were performed using iCell Cardiomyocytes^®^ (Cellular Dynamics International, Madison, WI, USA) derived from hiPSCs. A 98 % pure population of cardiomyocytes was supplied in a cryopreserved single-cell suspension. The cardiomyocytes were a mixture of electrically active atrial-, nodal- and ventricle-like myocytes. Cryopreserved cardiomyocytes were thawed and plated on fibronectin-coated (5 µg/cm^2^, 2 h at 37 °C) (Sigma-Aldrich, Steinheim, Germany) 6-well plates and 96-well plates using iCell Cardiomyocytes Plating Medium (iCell-PM) (Cellular Dynamics International, Madison, WI, USA). Two days post-plating, cells were cultured in iCell Cardiomyocytes Maintenance Medium (iCell-MM) (Cellular Dynamics International, Madison, WI, USA), with fresh media changed every 2 days.

### Test compound

A 10 mM stock solution (in DMSO) of DOX was purchased from Selleck chemicals. The stock solution was dispensed into small volume aliquots and stored at −80 °C. The drug dilutions were performed in iCell-MM at room temperature prior to each drug exposure.

### DOX exposure and cell sample collection for microarray studies

**Fig. 1:**
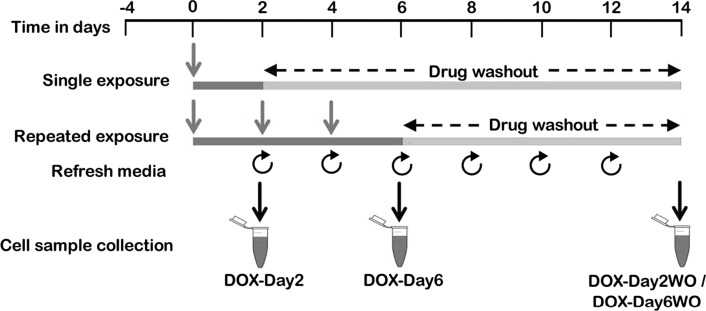
Experimental design for repeated drug exposure to hiPSC-CMs. Synchronously beating cardiomyocytes were given a single DOX exposure for 2 days or repeated DOX exposure for 6 days (the DOX-supplemented culture media was refreshed every 2 days). Following single and repeated drug exposure, the cells were cultured in drug-free culture media until day 14 (after the onset of drug exposure). During the drug washout, the culture medium was refreshed every 2 days. For microarray studies, cell samples were harvested on day 2, 6 and 14. This figure was reproduced from our previous report (Chaudhari et al. 2015)

A schematic representation of the experimental design is shown in Fig. 1. Briefly, the iCell cardiomyocytes were cultured in a 6-well plate at a density of 0.4 × 10^6^ cells per well. Four days post-plating, the synchronously beating cardiomyocytes were incubated with DOX at 156 nM for a 2-day exposure period (DOX-Day2) or three consecutive exposure periods of DOX for 6 days (DOX-Day6) (DOX supplemental media was refreshed every 48 h). Following DOX exposure for a 2-day washout (DOX-Day2WO) or a 6-day washout (DOX-Day6WO), cardiomyocytes were further cultivated in DOX-free iCell-MM until day 14 (from the start of the drug exposure). Non-treated control cells were cultured for 6 days in iCell-MM with DMSO as the solvent. The culture medium was refreshed every 2 days. The control, DOX-exposed and drug washout cell samples were harvested on day 2, 6 and 14, respectively, using QIAzol lysis reagent (Qiagen, Hilden, Germany). Total RNA, including non-coding miRNAs, were extracted from harvested cell samples and used for miRNA microarray hybridization and real-time PCR studies.

### Total RNA extraction

The total RNA, including non-coding miRNAs, were isolated from control, DOX-exposed and drug washout cardiomyocytes using the miRNeasy Mini Kit (Qiagen, Hilden, Germany) according to the manufacturer’s instructions. Concentration and purity of the isolated RNA was evaluated using a Nanodrop ND-1000 spectrophotometer (ND-1000, Thermo Fisher, Langenselbold, Germany). RNA integrity was confirmed using the Experion™ automated electrophoresis system (Bio-Rad, Munich, Germany).

### miRNA microarray labelling and hybridization

The FlashTag Biotin HSR RNA labelling Kit (Affymetrix, High Wycombe, United Kingdom) was used to label 500 ng of total RNA. The biotin labelled samples were hybridized to GeneChip miRNA 3.0 arrays (Affymetrix, High Wycombe, United Kingdom) using a hybridization cocktail. Microarray hybridization was performed in an Affymetrix GeneChip Hybridization Oven-645 for 16 h at 48 °C and 60 rpm. Hybridized arrays were washed and stained using the GeneChip HWS Kit (Affymetrix, High Wycombe, United Kingdom) and the fluidics station protocol (FS450_0002) on an Affymetrix GeneChip Fluidics Station-450. The stained arrays were scanned with an Affymetrix GeneChip Scanner-3000-7G, and quality of the scanned arrays was evaluated using Affymetrix GCOS software. The generated raw data files were used for data analysis.

### miRNA microarray data analysis

The miRNA expression data procured from the GeneChip^®^ miRNA 3.0 arrays were quantile normalized using the RMA approach with the Partek^®^ Genomics Suite^®^ software, version 6.6 (Copyright Partek Inc., St. Louis, MO, USA). The probe sets for 5787 miRNAs specific to *Homo sapiens* were selected further for statistical calculation. The differential expressions between groups were analysed for DOX-Day2 versus Control-Day2, DOX-Day6 versus Control-Day6, and DOX-Day2WO and DOX-Day6WO versus Control-Day14. Statistical calculations to determine significant genes were executed with the linear model implementation of the R Limma package followed by a Benjamini-Hochberg multiple test correction (1 % FDR). The miRNAs with a minimum fold change 1.8 and *p* value <0.05 were selected for further data analysis.

### Prediction of miRNA-gene targets

**Fig. 2:**
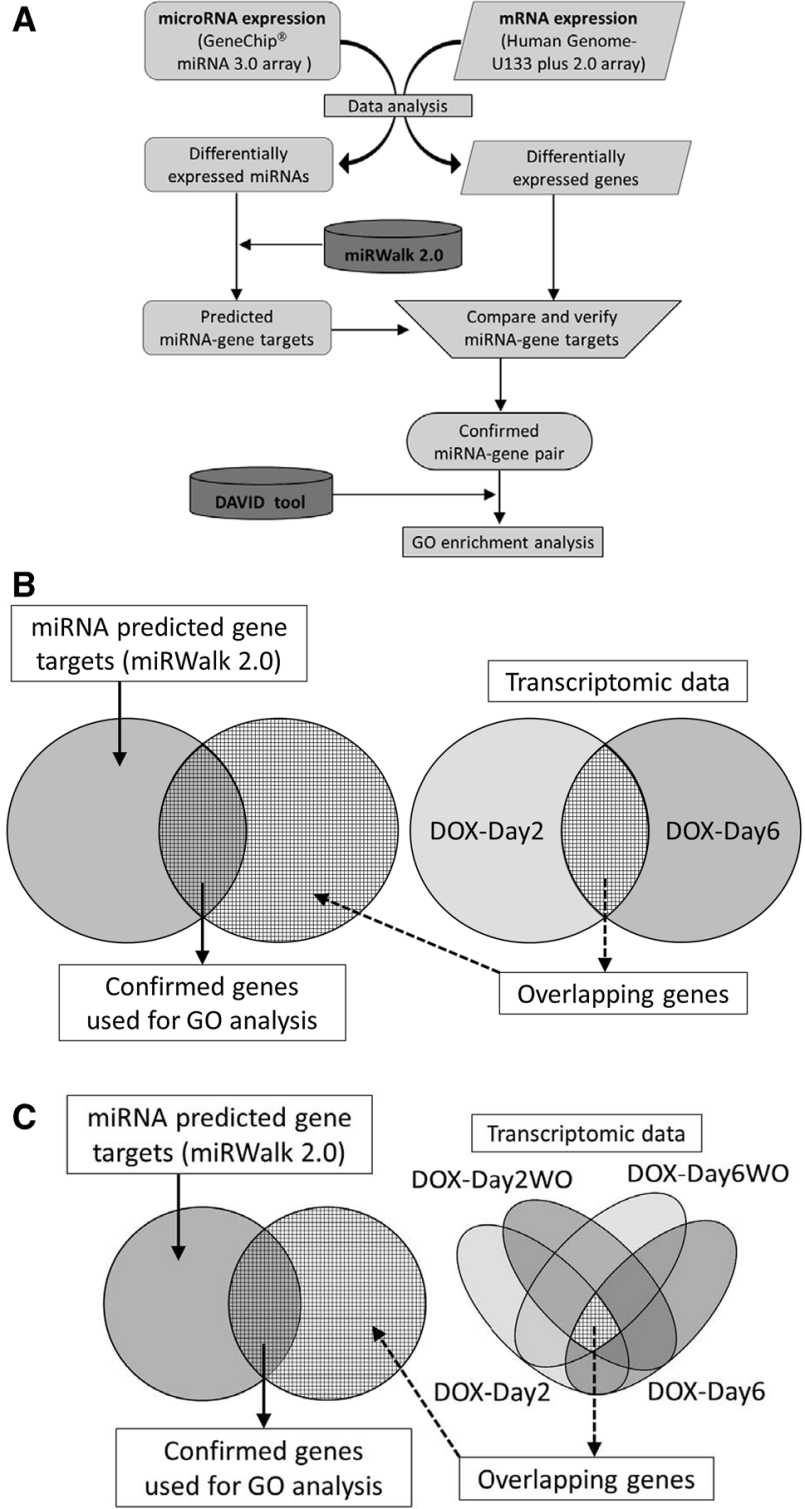
**a** Flow chart of the microarray data analysis used in this work. Differentially expressed miRNAs and their putative gene targets were verified with gene expression (mRNA) data subsequently the verified gene targets were used for the GO analysis. **b**, **c** Overlapping genes from the transcriptomic data matched with the miRNA gene targets predicted using miRWalk 2.0. Venn diagrams show that the predicted gene targets of the up-regulated miRNAs matched with the overlapping down-regulated genes and vice versa. Confirmed gene targets used for GO analysis

The gene target prediction of perturbed miRNAs was performed using the miRWalk 2.0 database (Dweep et al. 2011). Unlike currently available miRNA-gene target predictive tools, miRWalk 2.0 can identify putative miRNA binding sites not only in the 3′-UTR region but also in the promoter, the 5′-UTR and the CDS (amino acid coding sequence) regions of a gene. The miRWalk database is updated routinely and also provides information on validated miRNA binding sites in human genes. The predicted gene targets of the miRNAs were systematically compared and verified with our previously reported DOX transcriptomic data that contains differentially expressed genes (fold change of 2.0, FDR^®^ *p* value <0.05) for DOX-Day2, DOX-Day6, DOX-Day2WO and DOX-Day6WO groups (Chaudhari et al. 2015) (Fig. 2a). The predicted gene targets of the up-regulated miRNAs were verified with commonly down-regulated genes among the DOX-Day2 and DOX-Day6 groups, while the predicted gene targets of down-regulated miRNAs were confirmed by comparison with the commonly up-regulated genes between the DOX-Day2 and DOX-Day6 groups (Fig. 2b). Similarly, the predicted gene targets of the persistently up-regulated miRNAs were verified with prolonged down-regulated genes (Fig. 2c). Verified gene targets from the transcriptome data were used for Gene ontology (GO) analysis. The GO enrichment and KEGG pathway analyses were performed using the online Database for Annotation, Visualization and Integrated Discovery (DAVID) programme (Dennis et al. 2003).

### Quantitative real-time PCR (qPCR)

Using 500 ng of total RNA, cDNA synthesis was performed with the qScript™ microRNA cDNA Synthesis Kit (Quanta Biosciences, Gaithersburg, USA) following the manufacturer’s instructions. The cDNA was diluted fivefold with nuclease-free water, and 1 µl was used as a template for qPCR. The amplification of miRNA was performed using the PerfeCTa^®^ microRNA assay primer, the PerfeCTa^®^ Universal PCR primer and the PerfeCTa^®^ SYBR^®^ Green SuperMix, Low ROX™ Kit (Quanta Biosciences, Gaithersburg, USA) as per the manufacturer’s instructions. qPCR was carried out on an Applied Biosystems 7500 FAST Real-Time PCR System. Relative miRNA levels were calculated using the ΔΔ*C*_t_ method, and RNU6 was used as the miRNA PCR control.

### LDH leakage assay

Lactate dehydrogenase (LDH) is a widely used marker to evaluate the presence of damage and toxicity in cells and tissues. LDH is a cytosolic enzyme that is not normally discharged outside of the cell, but upon plasma membrane damage of cells, LDH is released into the cell culture medium or blood. Measurement of extracellular LDH release into the culture medium can be used to assay cellular toxicity. The iCell cardiomyocytes were seeded on a fibronectin-coated (5 µg/cm^2^, 2 h at 37 °C) 96-well plate at a cell density of 20 × 10^3^ per well. On day 4 post-seeding, cardiomyocytes were exposed to DOX at different concentrations. After 48 h of exposure, extracellular LDH activity was measured using the Thermo Scientific™ Pierce™ LDH Cytotoxicity Assay Kit according to the manufacturer’s instructions. Absorbance was measured at 490 nm using the Softmax Pro M5e 96-well plate reader (Molecular Devices, Sunnyvale, CA, USA). LDH activity was expressed as fold change versus control samples.

## Results

### Repeated exposure to DOX-induced cytotoxicity

**Fig. 3:**
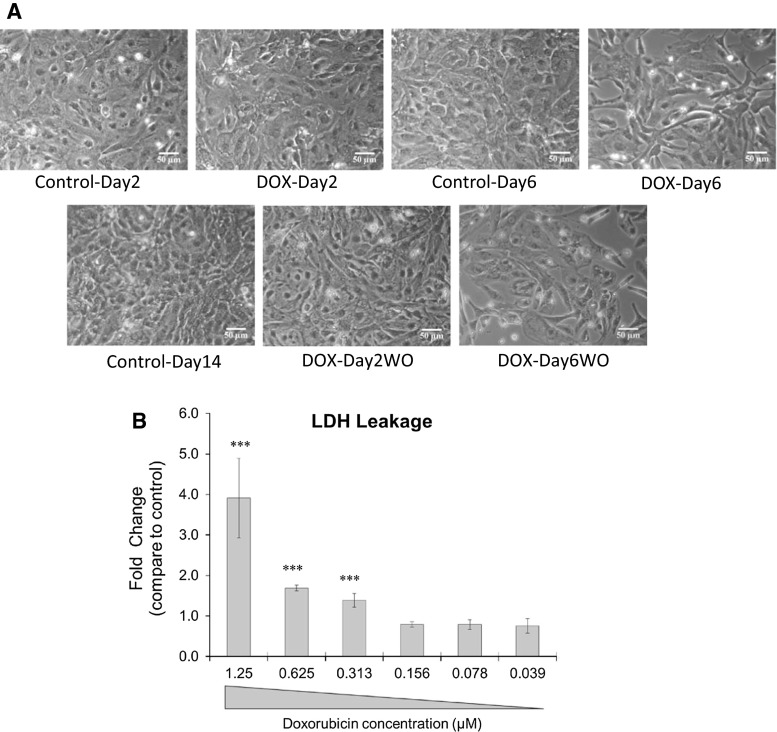
**a** Effect of DOX (156 nM) single and repeated exposure on cell viability. Cell images show that compared to controls, hiPSC-CM viability neither changed during single DOX exposure (DOX-Day2) nor during drug washout in DOX-Day2WO group. However, compared to controls, repeated exposure to DOX induced significant cytotoxicity and resulted in decreased cell viability in the DOX-Day6 group. During drug washout, slightly more cell death was observed in the DOX-Day6WO group compared to the DOX-Day6 group. *Scale bar* indicates 50 µm. **B,** DOX-induced cytotoxicity was assessed by LDH leakage assay. The hiPSC-CMs were incubated with DOX for 48 h at a concentration range of 0.039–1.25 µM. The enzymatic activity was analysed for the cellular release of LDH into the culture media. The *bar graph* shows mean values of fold change compared to controls, and the *error bars* indicate the standard deviation (*n* = 4). ***p value <0.001 versus control group

Similar to our previous report (Chaudhari et al. 2015), repeated exposure to DOX induced significant cytotoxicity at day 6 (DOX-Day6) compared to Control-Day6 cardiomyocytes (Fig. 3a). This result is indicative of a decrease in cell viability and ultimately cardiac cell loss. During drug washout, especially in repeatedly exposed cardiomyocytes (DOX-Day6WO), a slight, but not significant, decrease in cell viability was observed compared to the DOX-Day6 group. A single exposure of DOX did not induce a significant level of cytotoxicity in DOX-Day2 and DOX-Day2WO groups compared to the Control-Day2 and Control-Day14 cardiomyocytes, respectively. Additionally, no significant increase in LDH leakage was observed after 2 days of exposure to DOX at a concentration of 156 nM compared to controls (Fig. 3b). However, exposure to DOX at a concentration above 156 nM resulted in progressive increase in LDH release, which indicated the presence of cell membrane damage and cytotoxicity. These findings demonstrate that a 2-day exposure of DOX at 156 nM to cardiomyocytes could be used to identify early changes in miRNA expression levels under less-adverse cytotoxic conditions.

### miRNA profiling identified early DOX-responsive and persistently deregulated miRNAs

**Fig. 4:**
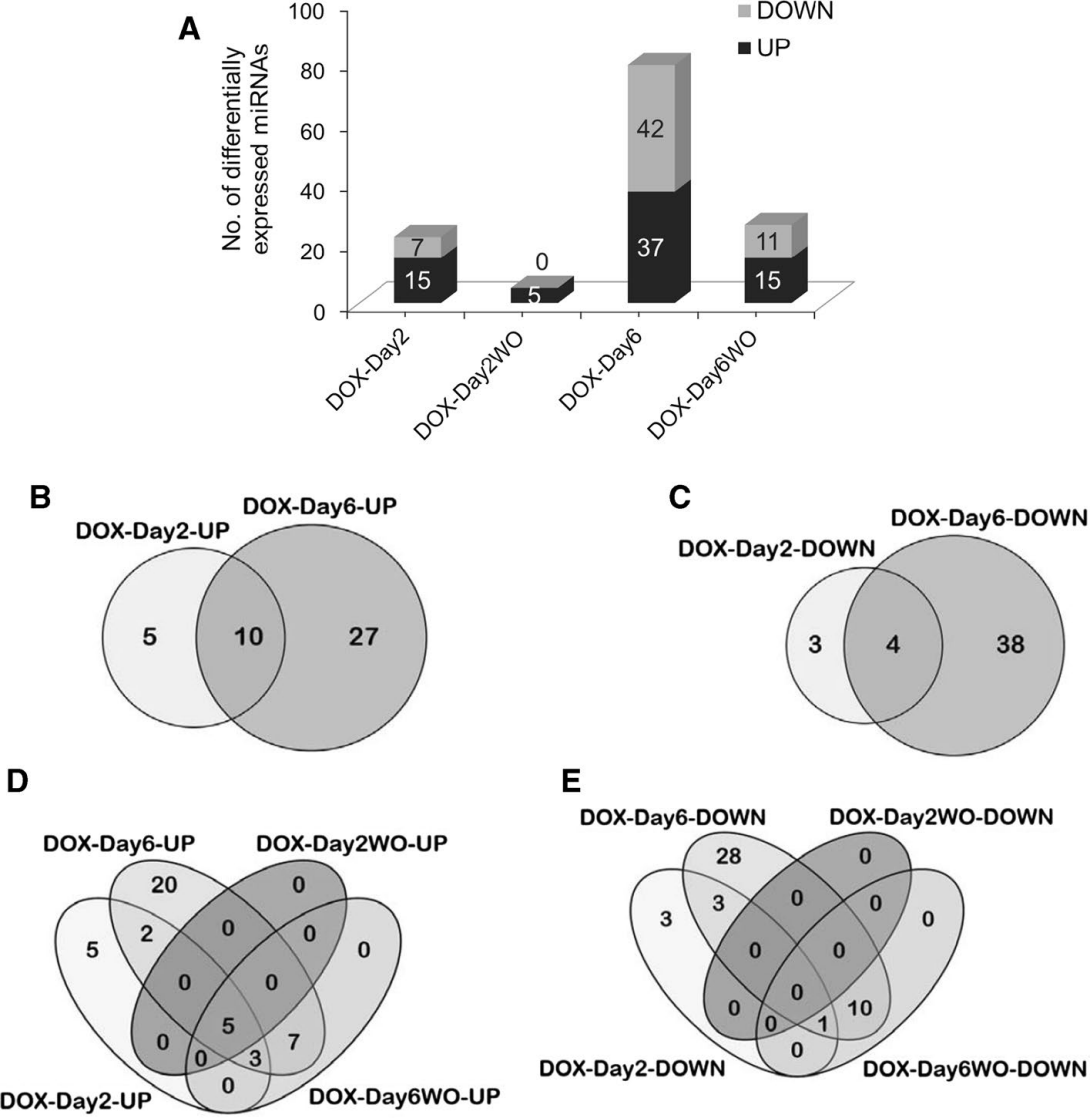
**a** Number of differentially expressed miRNAs in different experimental conditions. Three independent experiments were performed for miRNA microarray studies. Comparisons with respective control groups were performed to identify the DOX-induced differentially expressed miRNAs (with at least a fold change 1.8 and a *p* value <0.05 vs. control). The number of up- and down-regulated miRNAs is presented in the *bar graph*. The miRNAs, those did not deregulate during DOX exposure but showed deregulations during drug washout were omitted from washout groups before the data analysis. **b**, **c** Venn diagrams representing the number of commonly up- and down-regulated miRNAs among the DOX-Day2 and DOX-Day6 groups, respectively. **d**, **e** Venn diagrams illustrating number of overlapping up- and down-regulated miRNAs among the DOX-exposed and washout groups, respectively

The miRNA microarray data analysis resulted in the identification of several differentially expressed miRNAs in the DOX-treated and washout groups (Fig. 4a). A single exposure (DOX-Day2) and a repeated exposure (DOX-Day6) to DOX perturbed, in total, 21 (15 up-regulated and seven down-regulated) and 79 (37 up-regulated and 42 down-regulated) miRNAs, respectively. The five up-regulated miRNAs in the DOX-Day2WO group and the 26 miRNAs (15 up-regulated and 11 down-regulated) in the DOX-Day6WO group showed prolonged differential expression during drug washout. Differentially expressed miRNAs under different experimental conditions are provided in Supplemental Table S1. A Venn diagram analysis was performed for DOX-exposed and drug washout groups to identify commonly deregulated miRNAs. The analysis identified 14 (10 up-regulated and four down-regulated) commonly deregulated miRNAs between the DOX-Day2 and DOX-Day6 groups (Fig. 4b, c). These 14 deregulated miRNAs are indicative of an early response to DOX, and their fold change values are provided in Table 1. Among the DOX-exposed and drug washout groups, five miRNAs showed persistent up-regulation (Fig. 4d, and their fold change values are provided in Table 1), while no commonly down-regulated miRNAs were observed (Fig. 4e).

**Table 1:** Early and/or persistently deregulated miRNAs by DOX in hiPSC-CMs

miRNAs	DOX-Day2	DOX-Day6	DOX-Day2WO	DOX-Day6WO
miR-187-3p	20.7	66.6	14.1	33.6
miR-182-5p	10.9	16.9	3.0	10.2
miR-486-5p	5.4	3.6	2.1	2.8
miR-34a-3p	5.2	10.1	–	2.8
miR-486-3p	3.8	6.8	–	2.3
miR-212-3p	2.5	2.5	–	–
miR-4423-3p	2.3	7.2	2.35	4.0
miR-139-5p	2.0	1.9	–	–
miR-34c-3p	1.9	2.0	–	2.1
miR-34c-5p	1.8	4.5	2.22	2.3
miR-3911	−3.5	−3.7	–	–
miR-675-5p	−3.5	−9.2	–	−2.4
miR-4298	−2.0	−2.5	–	–
miR-1303	−1.8	−3.5	–	–

The fold change values were obtained using miRNA microarray data analysis (*n* = 3, *p* value <0.05 vs. control)

### Verified miRNA-gene targets enriched in cardiac and stress-associated GOs

Verified gene targets of each miRNA in Table 1 were first identified using the miRWalk 2.0 bioinformatics tool. The putative gene targets of the up-regulated miRNAs were matched with the identified DOX-induced differentially down-regulated genes at day 2, 6 and 14 as previously described (Chaudhari et al. 2015) (see Supplemental Table S2). Common confirmed genes were analysed for the enrichment of GO and KEGG pathways using the DAVID functional enrichment programme. This analysis revealed early DOX-affected cardiac processes, pathways and general toxic responses (Table 2). The down-regulated genes were mainly enriched for GO terms such as the sarcomere, muscle contraction and ion channel activity, and KEGG pathways such as cardiac muscle contraction, HCM, DCM and the MAPK signalling pathway. The putative target genes of the down-regulated miRNAs were matched with the up-regulated genes. Analysis of the common verified genes revealed enrichment for GO terms such as apoptosis, oxidative stress, inflammatory responses and the extracellular region (Table 2). Other GOs are provided in Supplemental Table S3. Verified gene targets of miR-187-3p, miR-486-5p, miR-34a, miR-212-3p, miR-34c-3p, miR-675-5p and miR-3911 were not enriched for GO terms related to cardiac and general toxicity responses. Similarly, verified gene targets of persistently up-regulated miRNAs were not enriched for cardiac-related GOs.

**Table 2:** Significantly enriched GOs of verified gene targets of early DOX-responsive miRNAs

Category	Term	Count	*p* value	Downregulated genes
miR-182-5p				
KEGG_PATHWAY	hsa04260:Cardiac muscle contraction	3	1.60E−02	*COX6A2*, *ATP1A2*, *MYH6*
miR-486-3p				
GOTERM_CC_FAT	GO:0030017~sarcomere	4	3.00E−03	*DES*, *LDB3*, *FHL2*, *MYH7*
GOTERM_BP_FAT	GO:0006936~muscle contraction	4	1.90E−02	*DES*, *MYH7*, *SCN5A*, *SGCA*
KEGG_PATHWAY	hsa05410:Hypertrophic cardiomyopathy (HCM)	3	3.20E−02	*DES*, *MYH7*, *SGCA*
GOTERM_BP_FAT	GO:0008016~regulation of heart contraction	3	3.30E−02	*DES*, *MYH7*, *SCN5A*
KEGG_PATHWAY	hsa05414:Dilated cardiomyopathy (DCM)	3	3.70E−02	*DES*, *MYH7*, *SGCA*
GOTERM_MF_FAT	GO:0005216~ion channel activity	5	4.70E−02	*KCNN2*, *ANO4*, *SCN5A*, *FXYD6*, *KCNK3*
miR-4423-3p				
KEGG_PATHWAY	hsa04260:Cardiac muscle contraction	3	1.20E−02	*MYL3*, *ATP1B4*, *CACNA2D2*
GOTERM_CC_FAT	GO:0014704~intercalated disc	2	3.00E−02	*NRAP*, *CTNNA3*
GOTERM_BP_FAT	GO:0007507~heart development	3	4.70E−02	*MYL3*, *FGF12*, *GJA5*
miR-139-3p				
GOTERM_CC_FAT	GO:0030017~sarcomere	4	2.20E−03	*ANK2*, *DMD*, *LDB3*, *MYH7*
GOTERM_MF_FAT	GO:0008307~structural constituent of muscle	3	4.20E−03	*DMD*, *MYH7*, *ASPH*
GOTERM_CC_FAT	GO:0005856~cytoskeleton	9	2.60E−02	*MAD2L1*, *ANK2*, *SPAG5*, *DMD*, *LDB3*, *KIF18A*, *CENPE*, *MYH7*, *TOP2A*
miR-34c-5p				
KEGG_PATHWAY	hsa04010:MAPK signalling pathway	5	8.20E−03	*IL1R1*, *RASGRP2*, *FGF12*, *CACNA2D2*, *MYC*
GOTERM_BP_FAT	GO:0006936~muscle contraction	4	1.30E−02	*MYOM2*, *ARG2*, *MYH6*, *SCN5A*
GOTERM_CC_FAT	GO:0030017~sarcomere	3	3.40E−02	*ANK2*, *FHL2*, *MYH6*

Category	Term	Count	*p* value	Up-regulated genes
miR-1303				
GOTERM_BP_FAT	GO:0006979~response to oxidative stress	3	4.80E−02	*EGFR*, *GPX1*, *OXR1*
miR-4298				
GOTERM_CC_FAT	GO:0044421~extracellular region part	12	3.10E−04	*PROM1*, *A2M*, *THBD*, *MASP1*, *SULF2*, *COL27A1*, *ACE2*, *MFGE8*, *GDF15*, *NRG1*, *RNPEP*, *APLP1*
GOTERM_BP_FAT	GO:0042981~regulation of apoptosis	8	2.50E−02	*DHRS2*, *EI24*, *BDNF*, *TNFRSF10B*, *TNFRSF10D*, *NRG1*, *GSTP1*, *TP53INP1*
GOTERM_BP_FAT	GO:0050727~regulation of inflammatory response	3	3.10E−02	*A2M*, *MASP1*, *ACE2*
GOTERM_BP_FAT	GO:0006916~anti-apoptosis	4	3.80E−02	*BDNF*, *TNFRSF10D*, *NRG1*, *GSTP1*
GOTERM_BP_FAT	GO:0002526~acute inflammatory response	3	4.90E−02	*A2M*, *MASP1*, *TRPV1*

The data show a significant enrichment of cardiac-related GOs and pathways for upregulated miRNAs, specifically, miR-182-5p, miR-486-3p, miR-4423-3p, miR-139-3p and miR-34c-5p, while down-regulated miRNAs, such as miR-1303 and miR-4298, were enriched for GOs related to stress and apoptosis

### Validation of deregulated miRNA expression using qPCR

**Fig. 5:**
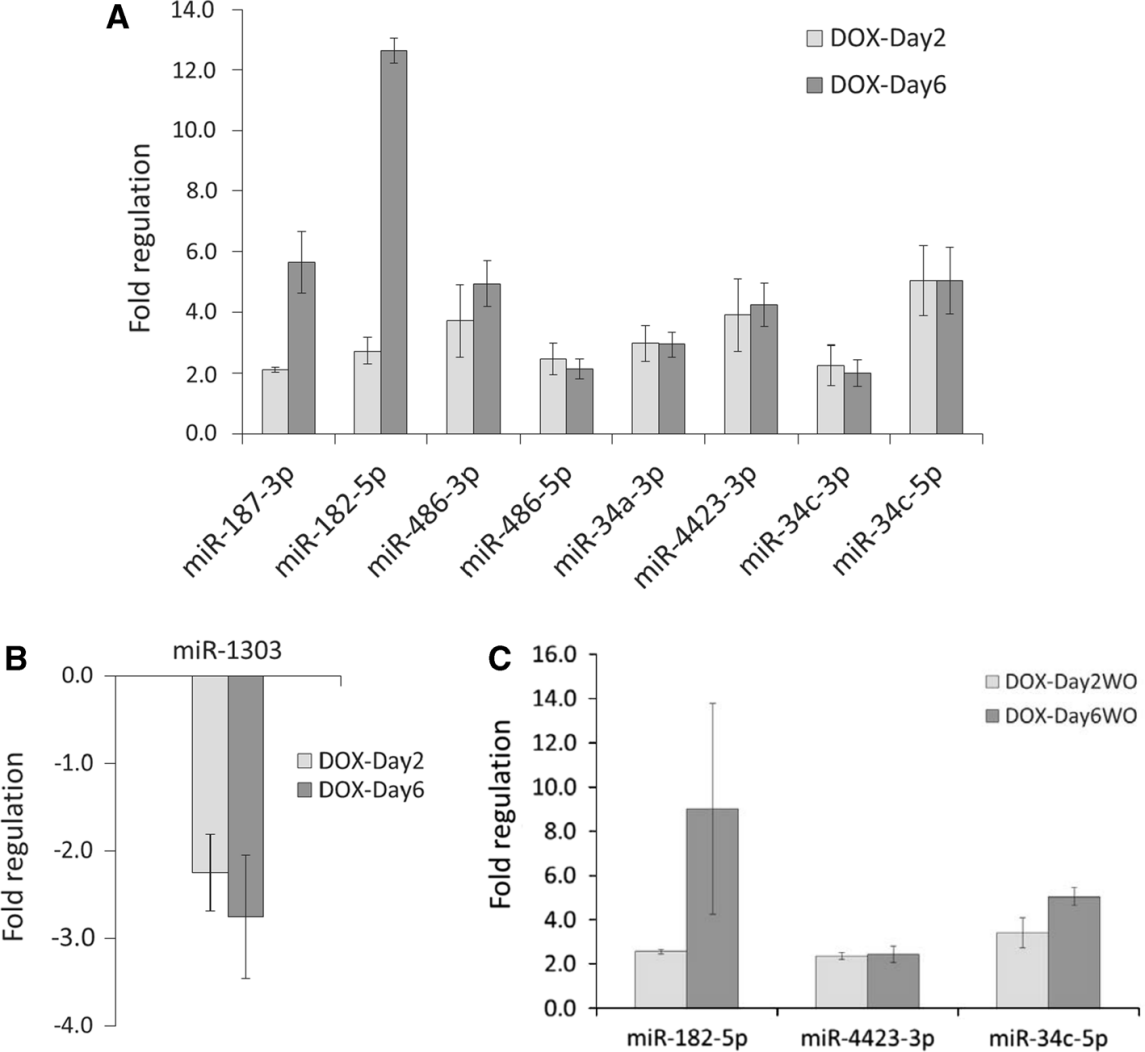
Quantitative real-time PCR (qPCR) validation of miRNA microarray data. The qPCR confirmed significant **a** up-regulation of eight miRNAs, **b** down-regulation of miR-1303 in both the DOX-Day2 and DOX-Day6 groups, and **c** prolonged up-regulation of miR-182-5p, miR-4423-3p and miR-34c-5p in the drug washout groups at day 14. The *bar graph* represents the fold regulation of miRNAs as the mean ± standard deviation (*n* = 3, *p* value <0.05 vs. control)

The 14 early DOX-responsive miRNAs and the five persistently up-regulated miRNAs were validated using qPCR (for primers sequences see supplemental Table S4). The qPCR data analysis confirmed deregulation of nine miRNAs (8 up and 1 down) in both DOX-Day2 and DOX-Day6 groups (Fig. 5a, b), and also confirmed the prolonged up-regulation of three miRNAs in the washout groups at day 14 (Fig. 5c).

## Discussion

High throughput miRNA profiling is progressively becoming an important screening tool to identify miRNAs that are responsive to drugs and environmental factors. Using this approach, in the present study, we identified early and prolonged DOX deregulated miRNAs that can be used to detect cardiotoxicants. Myocardial apoptosis is one of the main causes of progressive heart failure during DOX treatment. In the present study, repeated exposure to 156 nM DOX-induced substantial cytotoxicity in the DOX-Day6 group, which resulted in cell death and loss of cardiomyocytes. In addition, we also found that compared to controls and a single-DOX exposure group, hiPSC-CMs after repeated exposure to DOX showed arrhythmic beating (Chaudhari et al. 2015). Previous reports have shown that DOX induces myocardial apoptosis in animal models (Fisher et al. 2005; Sharov et al. 1996; Ueno et al. 2006). Increasing evidence suggests that apoptosis is crucially involved in the loss of cardiomyocytes in failing human hearts (Olivetti et al. 1997), myocarditis (Kawano et al. 1994) and myocardial infarction (Saraste et al. 1997). Ageing is also associated with a gradual decrease in cardiac performance and a loss of cardiomyocytes through apoptosis (Kwak 2013).

In this study, a miRNA microarray analysis revealed 14 early DOX-responsive miRNAs. Of these 14 miRNAs, nine returned to basal levels during drug washout, whereas the other five miRNAs showed persistent up-regulation during drug washout. These results suggest that the effect of DOX on the expression of the majority of miRNAs in cardiomyocytes is reversible if cardiomyocytes are only acutely exposed to DOX.

Validation of miRNA microarray data using qPCR confirmed deregulation of miR-187-3p, miR-182-5p, miR-486-3p, miR-486-5p, miR-34a-3p, miR-4423-3p, miR-34c-3p, miR-34c-5p and miR-1303 in both DOX-Day2 and DOX-Day6 groups. qPCR also confirmed the prolonged up-regulation of miR-182-5p, miR-4423-3p and miR-34c-5p in DOX-Day2WO and DOX-Day6WO groups. These identified deregulated miRNAs showed an early response to DOX that occurred before the induction of general cytotoxic biomarkers such as LDH. A 156 nM DOX treatment for 24 and 48 h did not induce a significant release of cardiac troponin T (cTnT) from hESC-derived cardiomyocytes in culture medium (Andersson et al. 2010; Holmgren et al. 2015). These observations indicate that miRNAs appear to be more sensitive toxicity signatures compared to LDH and other biomarkers such as cardiac troponin in DOX-exposed hiPSC-CMs. qPCR validated the use of the identified miRNAs in further investigations.

The miR-34 family members are involved in cardiac ageing, cardiac diseases and in cardiac apoptotic events. Recently, significant up-regulation of miR-34a has been reported in the aged hearts of mice (Boon et al. 2013), after myocardial infarction in mice (Lin et al. 2010) and after human dilated cardiomyopathy (Elzenaar et al. 2013). This up-regulation of miR-34a is associated with a loss of cardiomyocytes via apoptosis in both myocardial infarction patients and rats (Fan et al. 2013). Higher plasma levels of miR-34a may be a predictive indicator of heart failure after acute myocardial infarction in patients (Matsumoto et al. 2013) and may be a potential biomarker for coronary artery disease (Han et al. 2015). DOX-induced up-regulation of miR-34a has been reported recently in a mouse model (Desai et al. 2014). In agreement with these observations, our results show that DOX-induced up-regulation of miR-34a-3p expression can be correlated with increased apoptosis and decreased cell viability in repeatedly exposed cardiomyocytes (DOX-Day6). These results suggest a potential role of miR-34a-3p in early cytotoxic events in hiPSC-CMs.

Similarly, miR-34b and miR-34c (miR-34 family members) are up-regulated during rat cardiac hypertrophy (Feng et al. 2014), in the aged hearts of mice (Boon et al. 2013), in mouse hearts with myocardial infarction (Bernardo et al. 2012) and in diabetic ischaemic heart failure patients (Greco et al. 2012). DOX-induced up-regulation of miR-34b and miR-34c was reported in a mouse model (Desai et al. 2014) and in rat hearts (Vacchi-Suzzi et al. 2012). Consistent with these observations, our data demonstrated early up-regulation of miR-34c-3p and miR-34c-5p during DOX exposure. Furthermore, miR-34c-5p showed persistent up-regulation after the drug washout. Enriched GOs of verified gene targets of miR-34c-5p included those such as the sarcomere and muscle contraction and verified down-regulated gene targets including ion channels such as *KCNN2*, *SCN5A*, *CACNA2D2* and *KCNK3*. As previously shown, these findings can be linked to arrhythmic beating in cardiomyocytes during repeated exposure to DOX (Chaudhari et al. 2015). These observations suggest that up-regulation of miR-34c-3p and miR-34c-5p may be early indicators of cardiac dysfunction, the development of cardiac pathologies and future heart failure.

A higher expression level of miR-486-3p has been found in cats with hypertrophic cardiomyopathy (Weber et al. 2015) and in humans with DCM and HCM (Leptidis et al. 2013). Consistent with these reports, our present study shows an up-regulation of miR-486-3p in DOX-exposed cardiomyocytes. Additionally, the enriched GOs of the verified gene targets of miR-486-3p included those related to cardiac contractile function and ion channel activity. Also, the enriched KEGG pathways included HCM and DCM. Early up-regulation of miR-486-3p could lead to impaired cardiac function, and possibly to cardiomyopathies in hiPSC-CMs.

miR-486 is one of the highly enriched miRNA species in human and mouse hearts (Alexander et al. 2014). Overexpression of miR-486 reduces PTEN levels and activates PI3K/AKT signalling, which eventually leads to cardiac hypertrophy (Small et al. 2010b). Increased expression of miR-486-5p has been found in the plasma of acute myocardial infarction patients (Hsu et al. 2014) and also in cats with hypertrophic cardiomyopathy (Weber et al. 2015). Our findings showing a DOX-induced early up-regulation of miR-486-5p suggest that elevation of miR-486-5p may be considered as an early event in the development of cardiovascular diseases.

Among early up-regulated miRNAs, miR-187-3p showed the highest up-regulation in our miRNA microarray data analysis. Until now, no reports of miR-187-3p in heart failure and DOX-mediated cardiotoxicity have been observed. For the first time, we demonstrated that DOX caused a higher expression of miR-187-3p in human cardiomyocytes. Notably, miR-187-3p overexpressing T-lymphoma cells show resistance to DNA damaging agents such as DOX, cisplatin and cyclophosphamide (Yan et al. 2014). Thus, we may assume that miR-187-3p overexpression in cardiomyocytes is the result of DOX-induced DNA damage. However, more studies are needed to unveil the role of miR-187-3p in both cardiotoxicity and the development of heart failure.

An elevated level of miR-182 was found in patients with coronary disease (Taurino et al. 2010), dilated cardiomyopathy and chronic congestive heart failure (Zhu et al. 2013). DOX-induced DNA damage is an early event of lethal cardiac injury and cell death (L’Ecuyer et al. 2006). Moreover, increased levels of miR-182 impede the DNA repair process and increase genomic instability in cancer cell lines (Krishnan et al. 2013; Moskwa et al. 2011; Yao and Ventura 2011). Additionally, increased expression of miR-182 in melanoma cells after DOX treatment has been reported (Yan et al. 2012). Similarly, in our study, DOX induced the up-regulation of miR-182-5p in hiPSC-CMs, while verified gene targets enriched KEGG pathways such as cardiac muscle contraction. These observations suggest that miR-182-5p may be an initial indicator of cardiac injury and possibly DNA damage. The prolonged up-regulation of this miRNA may explain the mechanism of DOX-induced cardiac diseases and heart failure.

In the present study, we found that DOX induced an early and prolonged up-regulation of miR-4423-3p in hiPSC-CMs. Verified gene targets of miR-4423-3p were significantly enriched in both cardiac-specific GOs and KEGG pathways such as cardiac muscle contraction. Further investigation is needed to understand role of this miRNA in early cardiac damage, and more generally, in the progressive development of cardiac diseases. Our study also showed down-regulation of miRNAs particularly miR-1303 and miR-4298 after DOX exposure. In addition GO enrichment analysis showed that verified gene targets of miR-1303 and miR-4298 significantly enriched GOs associated with stress and apoptosis. However, role of these two miRNAs in development of cardiac pathogenesis or heart failure or ageing needs an in-depth investigation.

As we previously described (Chaudhari et al. 2015), a single exposure of 156 nM DOX compromised the beating heart rate without inducing cytotoxicity. In addition, a 156 nM concentration of DOX is within the therapeutic range of DOX. Repeated exposure to DOX (156 nM) induced substantial cytotoxicity in hiPSC-CMs. The nine validated miRNAs were significantly deregulated with a single exposure to DOX before the indication of the cytotoxicity marker LDH. In the present study, an early up-regulation of miR-34 family members was identified, which indicated that apoptosis could be a very early molecular event that induces cardiac cell loss in hiPSC-CMs after repeated exposure to DOX. Other early up-regulated miRNAs suggest a possible risk of cardiovascular diseases, such as cardiac hypertrophy and future heart failure. Prolonged up-regulation of three miRNAs suggests a risk of cardiac pathophysiology during the long-term recovery phase of DOX-exposed hiPSC-CMs. The DOX-induced miRNA deregulation in hiPSC-CMs is an indicative of complex molecular changes related to apoptosis, ageing, senescence, hypertrophy and other cardiac diseases, which ultimately progress towards cardiac dysfunction and heart failure. The early-deregulated miRNAs identified in this study might be beneficial for the identification and development of early genomic biomarkers of cardiotoxicity in humans and may also be useful for evaluating the risk of cardiac damage caused by potential therapeutic drugs and/or environmental factors with similar mechanism of action.

## Electronic supplementary material

Below is the link to the electronic supplementary material.

Supplementary material 1 (XLSX 21 kb)

Supplementary material 2 (XLSX 46 kb)

Supplementary material 3 (XLSX 401 kb)

Supplementary material 4 (DOCX 14 kb)
