# Supplementary material for: MicroRNAs as early toxicity signatures of doxorubicin in human-induced pluripotent stem cell-derived cardiomyocytes
Source: Arch Toxicol. 2016 Feb 3;90(12):3087–98. doi: 10.1007/s00204-016-1668-0 (PMC5104806; doi:10.1007/s00204-016-1668-0)
Supplement: Supplementary file 4 — Supplementary material 4 (DOCX 14 kb) [file 204_2016_1668_MOESM4_ESM.docx]

**Supplemental Table S4.** Primers used for qPCR validation of the different microRNAs deregulated by doxorubicin.

| **microRNA name (*Homo sapiens*)** | **Mature sequence** |
| --- | --- |
| miR-187-3p | UCGUGUCUUGUGUUGCAGCCGG |
| miR-182-5p | UUUGGCAAUGGUAGAACUCACACU |
| miR-486-5p | UCCUGUACUGAGCUGCCCCGAG |
| miR-34a-3p | CAAUCAGCAAGUAUACUGCCCU |
| miR-486-3p | CGGGGCAGCUCAGUACAGGAU |
| miR-212-3p | UAACAGUCUCCAGUCACGGCC |
| miR-4423-3p | AUAGGCACCAAAAAGCAACAA |
| miR-139-5p | UCUACAGUGCACGUGUCUCCAGU |
| miR-34c-3p | AAUCACUAACCACACGGCCAGG |
| miR-34c-5p | AGGCAGUGUAGUUAGCUGAUUGC |
| miR-3911 | UGUGUGGAUCCUGGAGGAGGCA |
| miR-675-5p | UGGUGCGGAGAGGGCCCACAGUG |
| miR-4298 | CUGGGACAGGAGGAGGAGGCAG |
| miR-1303 | UUUAGAGACGGGGUCUUGCUCU |
